# Insights into uncovered public health risks. The case of asthma attacks among archival workers: a cross-sectional study

**DOI:** 10.3389/fpubh.2024.1397236

**Published:** 2024-08-21

**Authors:** Liu Yang, Chen Xinting, Zhang Aie, Xu Ruiqi, Paulo Moreira, Dou Mei

**Affiliations:** ^1^Shandong Provincial Chronic Disease Hospital, Qingdao, China; ^2^School of Public Health, Qingdao University, Qingdao, China; ^3^Qilu Hospital, Shandong University, Jinan, China; ^4^The First Affiliated Hospital of Shandong First Medical University & Shandong Provincial Qianfoshan Hospital, International Healthcare Management Research and Development Centre (IHM-RDC), Jinan, China; ^5^Henan Normal University, School of Social Affairs, Xinxiang, China; ^6^Atlantica Instituto Universitario, Gestao em Saude, Oeiras, Portugal; ^7^Qingdao University Archives, Qingdao, China

**Keywords:** public health, asthma attacks, archival workers, chemical irritants, molds

## Abstract

**Objective:**

To ascertain the prevalence of asthma attacks among archivists and identify the associated occupational factors in this understudied professional population.

**Methods:**

We conducted a cross-sectional, questionnaire-based study among 1,002 archival workers. A multiple logistic regression was conducted to identify the association between asthma attacks and occupational exposures. The Strobe Protocol was applied.

**Results:**

999 workers were included in the final analysis with the asthma prevalence of 33.3%. Main factors associated with asthma attacks (OR [95% CI]) were the presence of chemically irritating odors (2.152 [1.532–3.024]), mold odors (1.747 [1.148–2.658]), and insects (1.409[1.041–1.907]). A significant synergistic effect was observed between chemical irritants and mold, the odds ratio was 7.098 (95% CI, 4.752–10.603).

**Conclusion:**

There was a high prevalence of asthma attacks among archival workers, an under-studied population. Chemical irritants, molds and insects were associated with their asthma attacks. Notably, this study’s data analysis has revealed a strong synergy (OR = 7.098) between chemical odors and molds in the workplace. While the existing international literature on this specific interaction remains somewhat limited, previous studies have already demonstrated the potential for chemical irritants, such as sulfur dioxide and ozone, to synergistically interact with inhalable allergens, including fungi, molds and dust mites. Consequently, this interaction seems to exacerbate asthma symptoms and perpetuate untreated exposure. Furthermore, in damp and damaged buildings, the presence of microbial components, such as cellular debris or spores released during fungal growth can trigger an inflammatory response, potentially served as a shared pathway for the development of asthma among individuals exposed to these hazardous factors.

## 1 Introduction

Asthma is a heterogeneous clinical syndrome that affects approximately 360 million people worldwide. Studies have found that up to 25% of adult asthma cases are work-related (1). The incidence does not appear to be decreasing (2), which leading to significant social and economic burdens. Recently, new cases of work related asthma due to workplace exposures in many sectors have been reported (3–5). Among all the workplaces, offices are not frequently associated with common agents for occupational asthma, office workers consequently remain a low risk of contracting occupational asthma (6, 7). Among all the workplaces, with relatively few exposure conditions associated with the incidence of occupational asthma, the risk of occupational asthma in the offices is low. However, A prevalence study conducted among office workers found totally 9.6% had doctor-diagnosed asthma (8). Anderson et al. (9) found that administrative support workers, including clerical workers and health service workers had significantly higher prevalence ratios (PR 1.5, 95%CI 1.2–1.9) of current asthma than prevalence in all industries. Thus, attention should be paid to the more specific occupational groups used to be simply classified as office workers.

Asthma can be triggered by factors such as exposure to allergens or irritants (10). A positive association between HDM allergens, dust, indoor air, mold, solvents and respiratory symptoms in office workers was reported (8, 11).

Related studies have shown a positive correlation between HDM allergens, dust, indoor air, mold, solvents and respiratory symptoms in office workers. The influencing factors of asthma are intricate, mainly attributed to genetic and environmental factors (12). Some researches have pointed out that there is also a certain amount of fungal pollution in different working environments, such as hospitals, nursing homes, museums and so on (13–15). When the fungus is exposed to a certain concentration it can cause asthma attacks in residents or staff (16, 17). Among them, archivists are susceptible to asthma due to the influence of working area and working mode.

Archive workers, a more specific occupational group as part of the office workers, besides dealing with relevant works in the office, closely expose to archive documents and document storage environment resulting. Most documents and files deposited in archives are made of paper, which are susceptible to chemical and biological damage. As a consequence of the exposed items degradation, VOCs can be formed from the paper itself (18), including acetic acid, formic acid, furfural, furfural, 4-hydroxy benzoic acid, 4-hydroxy acetophenone (19). Cladosporium, Aspergillus, and Penicillium species are almost ubiquitous in the archives (20), which induces allergic reactions and further developing of asthma (1, 21, 22). Exposure to biological allergens such as insect and microorganism is another crucial potential risk factor associated with incidence of asthma (23, 24). To the best of our knowledge, no studies have investigated asthma among archive workers.

Therefore, we sought to identify the prevalence and factors associated with asthma attacks in archival workers. In this study, a questionnaire-based study was conducted among archivists to investigate the associated factors concerning asthma attacks, and concurrently assessed potential interactions that may augment the risk of asthma attacks.

### 1.1 Contribution to the field

The evidence generated in this study suggests the need to further study and protect archivists as there is a strong synergy between chemical odors and molds in interaction with the potential of chemical irritants, such as sulfur dioxide and ozone, to synergistically interact with inhalable allergens, including fungi, molds and dust mites. Consequently, this interaction seems to exacerbate asthma symptom, perpetuate untreated exposure and trigger an inflammatory response potentially serving as a shared pathway for the development of asthma among individuals exposed to these hazardous factors (add here one more paragraph or two clarifying what can this paper contribute to knowledge and unknown aspects associated with the topic and the population under study, to be arranged in an independent section).

Additionally, the occupational health of archivists, who are the participants and executors of the preservation of important historical materials in countries and organizations, affects the sustainable development of archival undertakings. Archival workers often need to deal with all kinds of archival materials, including photo archives, physical archives and paper archives, etc., which plays a key role in the archives management work. These files may contain a variety of pathogenic microorganisms, such as bacteria, viruses, fungi and parasites, which may pose a potential risk of pathogenic infection to the archivists. A large number of occupational health studies have found that the human body will develop allergy symptoms, respiratory diseases and other health problems in the poor indoor environment.

At present, there are many studies based on the correlation between asthma and other occupations, but few studies on occupational risk factors for archivists, especially for the prevalence of asthma attacks in archivists, no evidence or correlation studies have been found. Therefore, the study of occupational hazard factors for archivists concerned in this study is a critical and ongoing topic, and its related research is of great significance for the protection of the health and safety of archival professionals.

## 2 Subjects and methods

### 2.1 Subjects

This cross-sectional, questionnaire-based study was conducted at the archives nationwide in China in a multi-center setting, including archives of enterprises and institutions (74.78%), national comprehensive archives (23.22%) and specialized archives (2.10%). Individuals who were currently employed in archive setting were included in the study. The questionnaire is available as Supplementary material.

#### 2.1.1 Sample size calculation

The results of the total work-life microsimulation conducted by Laditka (25) showed that 14.9% (CI 13.4–16.3) of those with low trigger exposure risk reported asthma at least once. We classified archivists as having a low risk of triggering asthma exposure and considered the prevalence of asthma among archivists to be approximately 14.9%, calculated according to the PASS software. Based on α = 0.05, δ = 0.03, *p* = 0.149, the total sample size required was calculated to be 573 cases. Considering the possibility of invalid samples in the questionnaire, the final sample size required was calculated to be 717 cases.

### 2.2 Methods

#### 2.2.1 Questionnaire

The questionnaires were sent to all eligible archivists in February 2022, and archivists were requested to fill out the questionnaires within 10 days. Here, we define “asthma attacks and exacerbation of asthma attacks among archivists after work” as asthma attacks. Data on the following personal factors were collected: sex (male, female), age (20–30 years, 31–40 years,41–50 years,51–65 years), education (below bachelor degree, bachelor, master’s degree or above), duration of employment (≤5 years,6–10 years,11–20 years, ≥21 years) and family history of the respiratory system. In order to explore archivists’ knowledge of occupational hazard factors, we also collected the data: whether knowing the effective protection measures to risk factors in the archival profession and well protection in work according to the professional protection files? The response to each is either “yes” or “no.” Furthermore, work-related factors were gathered: archivists’ average frequency of contacting paper files at work (times per day) ≤1, 1–2, 3–4, ≥5. And the strict separation of the working areas from archives warehouses, dampness in the working area, chemically irritating odor in working areas, mold odor in working areas, insects (roaches, ants, tobacco beetle and dust mite etc.) in working area. The answer options in each question are dichotomous (yes or no). To further adjust for confounding factors, the self-administered questionnaire asked about the protection to adverse factors related to archival work, i.e., legislation of protective measures of risk factors in the archival profession, having equipment for occupational protection, training on occupational protections of archives. Finally, subjects were asked to answer if they had asthma or more frequent asthma attacks at work (especially when in contact with archival entities), including questions about asthma symptoms, namely wheezing, chest tightness or shortness of breath which were questions in questionnaire. Responses range from always, frequently, occasionally, never. The data collected were conducted in an anonymous fashion. Ethical approval was obtained from XXX [Anonymized by request from JOEM]. Electronic informed consent was obtained for each participant.

### 2.3 Statistical analysis

All statistical analysis was performed using SPSS 26.0. Initially, associations between personal factors (age, sex, duration of employment, family history) and asthma attacks were analyzed by Chi-square test or the Mann–Whitney-U test. Next, the association between the related factors in archival work, i.e., strict separation of working areas from archives warehouses, ventilation and its average time in warehouse, temperature and humidity of the warehouse in summer, chemically irritating odor, mold odor and insects (roaches, ants, tobacco beetle and dust mite etc) in working areas and asthma attacks were also assessed by Chi-square test. Thereby, logistic regression was conducted according to the *p* value. For the first selection of associated factors, univariate logistic regression analysis was performed. Subsequently, multiple logistic regression analysis was performed to assess independent association, in which the presence of asthma attacks was the objective variable and the associated factors that showed significant associations in the univariate analysis were the explanation variables. For the variables with a *p* value <0.05 in the univariable analysis were entered into the multiple logistic regression model. The interaction between the chemically irritating odors and mold odors was examined in the logistic regression model. Statistical significance was set at *p* < 0.05.

The Strobe Protocol for Cross-Sectional studies was applied.

## 3 Results

### 3.1 Basic characteristics of the subjects

A total of 1,002 people submitted questionnaires, of which 999 were valid and included in the final analysis, with an effective rate of 99.7%. The gender, age, education, and duration of employment of the respondents substantially matched the statistics of the National Bureau of Statistics 2021. As shown in Table 1, individuals with asthma attacks accounted for 33%, of which 67% were female. Duration of employment were less than 10 years for 45% (*n* = 453) of subjects and 11 years or more for 55% (*n* = 556). Approximately 66% of those archivists with family history of the respiratory system had asthma attacks (Table 1).

**Table 1:** Basic characteristics of the subjects by asthma attacks (*n* = 999) [*n* (%)].

	Total (*n* = 999)	Non-asthma attacks (*n* = 666)	Asthma attacks (*n* = 333)	*p*-value
Gender				
Female	722	499 (74.9)	223 (67)	0.008
Male	277	167 (25.1)	110 (33)	
Age (years)				0.008^a^
≤40	286	211 (31.7)	75 (22.5)	
41–50	413	265 (39.8)	148 (44.4)	
≥51	300	190 (28.5)	110 (33.0)	
Education				0.515
Below bachelor degree	157	106 (15.9)	51 (15.3)	
Bachelor	631	413 (62.0)	218 (65.5)	
Master’s degree or above	211	147 (22.1)	64 (19.2)	
Duration of employment (years)				
≤5	273	208 (31.2)	65 (19.5)	<0.001
6–10	180	124 (18.6)	56 (16.8)	
11–20	274	176 (26.4)	98 (29.4)	
≥21	272	158 (23.7)	114 (34.2)	
The average frequency of contacting paper files at work (times/day)				
≤1	154	110 (16.5)	44 (13.2)	0.031
1–2	313	208 (31.2)	105 (31.5)	
3–4	220	158 (23.7)	62 (18.6)	
≥5	312	190 (28.5)	122 (36.6)	
Family history of the respiratory system	41	14 (2.1)	27 (8.1)	<0.001
Exercise regularly	673	452 (67.9)	221 (66.4)	0.633

a Mann–Whitney test.

### 3.2 Work-related factors

Archive’s daily sanitary measures and status in terms of asthma attacks in archivists are shown in Table 2. The archivists who have strict separation of the working areas from archives’ warehouses tended to respond that they suffered less asthma attacks, and many individuals respond that the occurrence of dampness, pungent chemical odor, mold odor as well as the harmful insects in the working area were significantly associated with the asthma attacks among them. Archives’ protective measures thought to be associated with asthma attacks were also assessed, the results of which were shown in Table 3. Those archivists having documentation of archival occupational risk factors in the workplace and achieving standardized protection at work were prone to have a lower prevalence of asthma attacks. As archival workers are exposed to various hazards in the workplace, it is essential for them to take appropriate protective measures. However, many archives fail to raise awareness of the dangers present in the workplace. Only 135 archivists participating in the study have had professional protection protocols in place. This study data indicates that chemically irritating odors, mold odors, and insects in the workplace are correlated with asthma attacks amongst the population studied. Therefore, it suggests that the workplace environment plays a key role in the occurrence of asthma attacks, and achieving standardized protection at work is prone to promote a lower prevalence of asthma attacks in the context studied.

**Table 2:** Worker-related factors in archival work [*n* (%)].

	Non-asthma attacks (*n* = 666)	Asthma attacks (*n* = 333)	*p*-value
Frequency of cleaning in warehouses (times/month)			0.039
<1	139 (20.9)	74 (22.2)	
1	196 (29.4)	92 (27.6)	
2–3	177 (26.6)	67 (20.1)	
≥4	154 (23.1)	100 (30.0)	
Strict separation of the working areas from archives warehouses	530 (79.6)	230 (69.1)	<0.001
dampness in the working area	340 (51.1)	229 (68.8)	<0.001
Ventilation in warehouses			0.407
Power ventilation	184 (27.6)	105 (31.5)	
Natural ventilation	372 (55.9)	179 (53.8)	
Power and natural ventilation	110 (16.5)	49 (14.7)	
Average ventilation time of warehouses (hour/day)			0.184
Never	132 (19.8)	76 (22.8)	
1	210 (31.5)	83 (24.9)	
1–2	147 (22.1)	79 (23.7)	
≥2	177 (26.6)	95 (28.5)	
Warehouse temperature in summer (°C)			0.147
14–24	491 (73.7)	231 (69.4)	
>24	175 (26.3)	102 (30.6)	
Warehouse humidity in summer (%)			0.684
<45%	225 (33.8)	119 (35.7)	
45–60%	395 (59.3)	195 (58.6)	
>60%	46 (6.9)	19 (5.7)	
Pungent chemical odor in working areas	96 (14.4)	118 (35.4)	<0.001
Mold odor in working areas (except warehouses)	263 (39.5)	226 (67.9)	<0.001
Mold odor in archives warehouses	294 (44.1)	241 (72.4)	<0.001
Insects in working area	257 (38.6)	192 (57.7)	<0.001

**Table 3:** Protection to adverse factors related to archival work (*n* = 999) [*n* (%)].

	Non-asthma (*n*%)	Asthma (*n* %)	*p*-value
Legislation of protective measures of risk factors in the archival profession	117 (17.6)	42 (12.6)	0.044
Knowing the effective protection measures to risk factors in the archival profession	314 (47.1)	136 (40.8)	0.059
Well protection in work according to the professional protection files^※^	105 (89.7)	30 (71.4)	0.004

^※^Archivists whose workplace have professional protection files.

### 3.3 Factors associated with asthma attacks

The univariate and multivariate analysis are summarized in Table 4. In the multivariate logistic regression analysis, sex, working years, strict separation of the working areas from archives, mold odor, chemically irritating odor and insects in the workplaces were significantly associated with the incidence of asthma attacks (*p* < 0.05). Workers who had work experiences≥21 years (OR, 95%CI: 2.116, 1.420 ~ 3.153) had the odds of developing asthma attacks 1.1 times more than workers who had work experiences between 6 and 10 years. Workers without strict separation of the working areas from archives warehouses were 0.522 times more likely to develop asthma attacks (OR, 95%CI: 1.522, 1.096 ~ 2.113). Workers who found mold odor in working areas as well as warehouses had a higher risk of developing asthma attacks (OR, 95%CI: 1.747, 1.148 ~ 2.658; 1.666, 1.084 ~ 2.561 separately). Workers who found chemically irritating odor in working areas were 1.152 times more likely to develop asthma attacks (OR, 95%CI: 2.152, 1.532 ~ 3.024). Workers who found insects in working areas were 0.409 times more likely to develop asthma attacks (OR, 95%CI: 1.409, 1.041 ~ 1.907).

**Table 4:** Factors associated with asthma attacks among archivists in univariate and multivariable analyses.

Characteristic	Univariable			Multivariable		
	Odds radio	95%CI	*p*-value	Odds radio	95%CI	*p*-value
Gender						
Female	Ref			Ref		
Male	1.474	1.105–1.966	0.008	1.665	1.211–2.289	0.002
Family history of respiratory system	4.109	1.909–8.030	<0.001	3.928	1.908–8.085	<0.001
Working years in archives Department (years)			<0.001			0.003
≤5	Ref			Ref		
6–10	1.445	0.949–2.201	0.086	1.315	0.835–2.071	0.238
11–20	1.782	1.228–2.585	0.002	1.522	1.018–2.276	0.041
≥21	2.309	1.598–3.337	<0.001	2.115	1.419–3.152	<0.001
Strict separation of the working areas from archives warehouses	0.573	1.294–2.354	<0.001	1.523	1.097–2.115	0.012
Mold odor in working areas (except warehouses)	3.236	2.452–4.272	<0.001	1.738	1.14– 2.65	0.010
Mold odor in archives warehouses	3.315	2.493–4.407	<0.001	1.651	1.068–2.553	0.024
Chemical irritating odor in working areas	3.259	2.386–4.451	<0.001	2.145	1.526–3.017	<0.001
Insects in working area	2.167	1.658–2.832	<0.001	1.395	1.019–1.909	0.038
Dampness in the working area	2.111	1.600–2.785	<0.001	1.042	0.743–1.461	0.813

**Figure 1:**
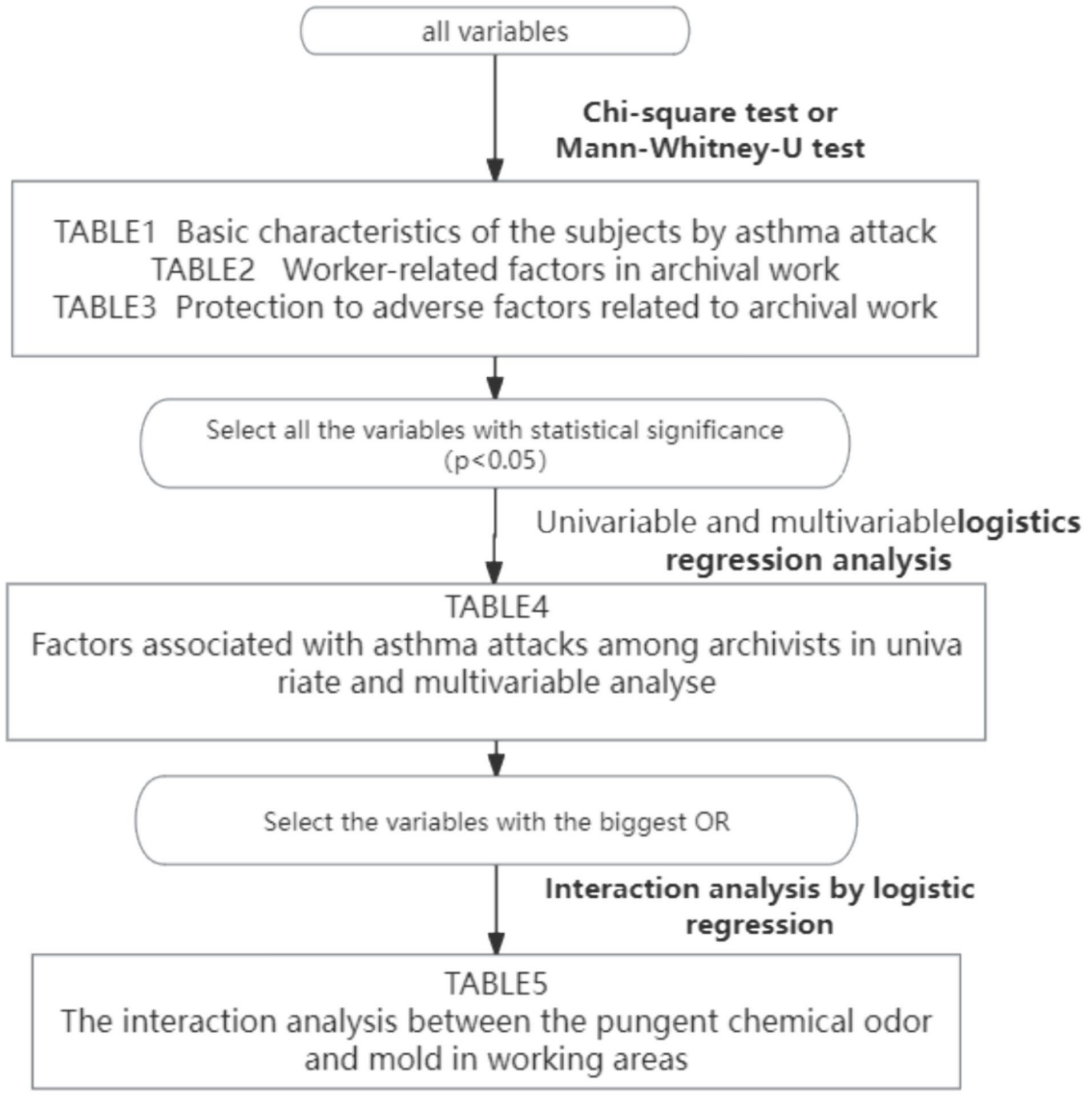
Program flowchart.

Table 5 shows the results of analysis in which we tested for interactions between chemically irritating odor and mold odor in working areas for asthma attacks. A significant synergistic effect was observed between chemical irritants and molds, the odds ratio was 7.098 (95% CI, 4.752–10.603). A program flowchart is presented in Figure 1.

**Table 5:** The interaction analysis between the pungent chemical odor and mold in working areas.

Factors	Number of subjects	Odds radio	95%CI	*p*-value
Chemically irritating odor (−) and mold odor (−)	451	Ref		
Chemically irritating odor (−) and mold odor (+)	59	2.345	1.310–4.199	0.004
Chemically irritating odor (+) and mold odor (−)	334	2.671	1.935–3.685	<0.001
Chemically irritating odor (+) and mold odor (+)	155	7.098	4.752–10.603	<0.001

## 4 Discussion

This study found that the prevalence of asthma attacks was 33.3%, higher among male archivists. The existence of chemically irritating odors and moldy smells within the work environment were associated with higher asthma attacks. We also found a significant synergistic effect between the two risk factors. To the best of our knowledge, this is the first multicenter study focusing specifically on the risk factors related to asthma in archivists and provides a fresh perspective on occupational asthma.

The present study identified a significant association between the presence of chemically irritating odors in archival workplaces and high asthma attacks among archivists. Occupational hazards for archivists primarily stem from indoor air pollution, including conventional indoor chemical pollutants such as formaldehyde, sulfur dioxide, volatile organic compound (26). Additionally, there are archival-specific chemical pollutants such as acetic acid, hydrogen sulfide, ethylene oxide, sulfuryl fluoride, furfural, and other compounds (19). It has been reported that exposure to ozone and sulfur dioxide has deleterious effects on immune competent cells and airway responsiveness (27). Owing to its potential to sensitize airway inflammation, ozone exhibits a propensity to induce various respiratory ailments, encompassing coughing and wheezing (28). It has been extensively elucidated that elevated ozone levels have an inflammatory impact on the respiratory system, thereby contributing to the progression of asthma (29). Interestingly, a study revealed a negative correlation between low-to-moderate atmospheric ozone levels and hospital visits by asthma patients (30). However, the measured median ozone concentration in office environments is 9.04 μg/m^3^ (31), which were consistent with this study’s results. A noteworthy association between the frequency of printer usage (exceeding seven times per day) and the occurrence of asthma attack was demonstrated in our study. A study conducted in Estonia, with participation from over 50,000 adults, have revealed a significant association between exposure ranging from low to moderate levels of indoor air pollutants and asthma (OR = 1.88, 95%CI 1.48 ~ 2.37) (22). Inhalation of VOCs, in particular, has been implicated in various adverse health effects (32), and their role in triggering asthma is well-documented. VOCs can activate the immune system, cause oxidative stress, and interact with some allergens (33). Metabolites of VOCs have also been found to be correlated with markers of oxidative stress, which are associated with lung function parameters (33–35). Furthermore, multiple studies have reported a connection between exposure to formaldehyde and the development of asthma and asthma symptoms in adults. Formaldehyde, as a respiratory irritant, exerts its effects by inducing inflammation of the airway mucosa and eliciting an inflammatory response via cytokines produced by Th2 cells (34). Additionally, transient decreases in lung function have been attributed to formaldehyde exposure (36).

In addition, this study has indicated mold as an associated factor for asthma among archivists, exhibiting a correlated escalation of 65.1 and 73.8% in warehouses and workplaces, respectively. Molds, being a potent allergens, can trigger allergic reactions, provoke inflammatory responses and augment the susceptibility to asthma via the emission of VOCs (26). A study has revealed a pronounced elevation of Asthma-COPD Overlap Syndrome (ACOS) associated with occupational exposure to mold odor, with an odds ratio (OR) of 3.43(95%CI 1.04–11.29) (37). Although limited research has been conducted among office workers, previous studies have consistently reported a positive correlation between mold odor and adult individuals in residential settings (38–40). Furthermore, a heightened susceptibility to asthma was detected in relation to occupational exposure to mold odor, as opposed to exposure within the confines of one’s abode (37). This could be potentially elucidated by the more pervasive prevalence of mold issues in archives, coupled with a tendency for individuals to expeditiously remedy any mold-related problems within their own dwellings. Another biological factor encountered in the workplace, i.e., dust mites and cockroaches, may contribute to an elevated risk of asthma by 39.5%. A recent study revealed that dust mite allergen concentration of 10 μg g-1 has been proposed as the threshold for asthma development. While the levels of Der p 1 and Der f 1 allergens in dust samples collected from offices in Malaysia were found to be as high as 556 ng/g and 658 ng/g, respectively (8). Our findings align with the previous study, as we have observed an increased asthma attacks in connection with exposure to dust mites and cockroaches (41). Hence, it is crucial to accord primacy to the eradication of dust particles and the implementation of sterilization protocols within archival repositories.

Notably, our data analysis has revealed a strong synergy (OR = 7.098) between chemical odors and molds in the workplace. While the existing literature on this specific interaction remains somewhat limited, previous studies have already demonstrated the potential for chemical irritants, such as sulfur dioxide and ozone, to synergistically interact with inhalable allergens, including fungi, molds and dust mites. Consequently, this interaction serves to exacerbate asthma symptoms and perpetuate untreated exposure (42). Furthermore, in damp and damaged buildings, the presence of microbial components, such as cellular debris or spores released during fungal growth can trigger an inflammatory response, potentially served as a shared pathway for the development of asthma among individuals exposed to these hazardous factors.

Interestingly, contrary to findings from other studies that reported a higher prevalence of asthma among women than men, this study revealed that the prevalence of asthma among archivists was 1.28 times higher among male workers. Stratified analysis by gender demonstrates significantly higher odds ratios (ORs) for males in both chemical and biological factors. More precisely, the presence of chemically irritating odors and biological agents, such as molds and dust mites, in the archival work environment is more robustly correlated with declining pulmonary function in males. This finding in concordance with a previous study carried out in Italy (43).

In conclusion, this survey revealed that approximately one-third of archivists experienced asthma attacks. Chemically irritating odor, Mold odor and insects in the workplaces are associated with asthma attacks. Moreover, chemically irritating gases and molds in the archival workplace were highly associated with asthma attack, with a significant interaction between them. However, the health relevance and mechanism of the work-related exposure in archives need to be further explored by more detailed assessments.

### 4.1 Strengths and limitations

There are several strengths in this study. First, the participation of archivists from various types of archives nationwide, who exhibit higher levels of compliance and consistency in their education and job type, significantly enhances the credibility and generalizability of our findings. Consequently, it is reasonable to extrapolate the results to a broader population of office workers. Second, given the intricate compositions, relatively low concentrations and inherent difficulty in precise measurement of various factors within the work environment, pertinent information was gathered through the employment of a questionnaire-based approach. Hence, employing a questionnaire to gauge the overall extent of exposure among archivists represents a judicious methodology within this framework. The study revised and adopted recent trends in healthcare research and related challenges (44–68).

Also, there are some limitations. First, as was the case for most of the previous studies, this was a cross-sectional study. Therefore, the causality between the asthma attacks and the associated factors were not clarified in this study. Second, current study surveyed subjects via a self-administered questionnaire, but the reliability and validity of this questionnaire was not tested. Second, the lack of quantification pertaining to the exposures presents a notable challenge in elucidating potential mechanisms of action or dose–response relationships. Third, similar to other large-scale population-based surveys, the diagnosis of asthma was based mainly on a standardized questionnaire, which could potentially have led to the misclassification of some pulmonary diseases.

### 4.2 Further research

Current study surveyed subjects via a self-administered questionnaire, but the reliability and validity of this questionnaire were not tested, especially the diagnosis of asthma. Therefore, future research is expected to add some questions, such as the doctor diagnosed asthma, asthma medication, etc. In addition, as mentioned above, the causality between asthma attacks and the associated factors was not clarified in this study. Clearly, future longitudinal studies are necessary to address this issue. Finally, concerning measurement of exposure-related factors. Microorganisms, dust particles, etc. in the archive working environment can be measured using more accurate measurement techniques or culture methods.

## Data Availability

The original contributions presented in the study are included in the article/Supplementary material, further inquiries can be directed to the corresponding authors.
