# Supplementary material for: Insights into uncovered public health risks. The case of asthma attacks among archival workers: a cross-sectional study
Source: Front Public Health. 2024 Aug 21;12:1397236. doi: 10.3389/fpubh.2024.1397236 (PMC11371701; doi:10.3389/fpubh.2024.1397236)
Supplement: Supplementary file 1 [file Data_Sheet_1.docx]

**Questionnaire on occupational hazard factors for archivists**

ⅠBasic information (questions 1~28)

1. Work place

- Shandong Province
- Qinghai Province
- Chongqing City
- Ningxia Hui Autonomous Region
- Other provinces and cities except the above

1. Your gender

- Man
- Woman

1. Your age

- ≤ 30 years old
- 31- -40 years old
- 41- -50 years old
- 51 years old

1. Your educational level

- Doctor’s degree
- Master’s degree
- Bachelor’s degree
- Junior college
- Below Junior college

1. Do you wear your glasses at work

- Yes
- No

1. Do you usually take physical exercise consciously?

- Yes
- No

1. What are your usual exercise methods ?[multiple choice]

- Jogging
- Walking
- Ball games
- Stretching exercise
- Strength training
- Others

1. Your work place

- The National Comprehensive Archives
- Enterprise unit archives
- Public institution archives
- Special archives, such as urban construction archives, photo archives, etc

1. Working years in the archives department

- ≤ 5 years
- 6-10 years
- 11-20 years
- 21-29 years
- ≥ 30 years

1. Average exposure frequency to archival entities at work

- ≥ 5 times per day
- 3-4 times per day in average
- 1-2 times per day in average
- 1 time per day in average
- Never

1. Protection procedures when exposing in archival entities include [multiple choice]

- Wear a mask
- Wear gloves
- Wear a hat
- Wear isolation clothes
- Others
- No

1. Building year of the current office space and warehouse

- Before 1970 year
- 1971-2000 year
- 2001-2010 year
- After 2010 year

1. Strict separation of the office area and the file warehouse

- Yes
- No

1. Floor of the archives warehouse

- Basement
- First floor (no basement)
- First floor (with basement)
- Second floor and above (excluding the top floor)
- Top floor

1. Ambient temperature of the warehouse in summer

- 14-24℃
- 24℃

1. Relative humidity of the warehouse in summer

- 45%
- 45-60%
- 60%

1. Ventilation mode of warehouse

- Power ventilation (such as fresh air system, ventilation fan, etc)
- Natural ventilation (such as opening the window)
- Power ventilation and natural ventilation

1. Average ventilation time of the warehouse

- ≥ 2 Hours Per Day
- 1-2 Hours Per Day
- 1 Hour Per Day
- Never

1. Daily cleaning frequency in warehouse

- ≥ 4 times per month
- 2-3 times per month
- 1 time per month

1. Do you aware of the occupational hazard factors involved in archival work?

- Knows a lot
- Knows a little
- No

1. Do you know the source of occupational hazards in archival work?

- Yes
- No

1. In your opinion, the occupational hazard factors of archivists mainly come from[multiple choice]

- Release of harmful chemicals of decoration materials in the office environment
- Release of harmful substances during the storage process of the archival entity
- Residual use of disinfectant in the process of file disinfection or after disinfection
- Harmful chemicals released during the operation of printers, photocopiers and other electronic equipment in daily work
- Ionizing radiation generated in the operation process of computers, photocopiers and other electronic devices in daily work
- Particulate matter in the warehouse, such as pm10, pm2.5, etc
- Radon
- Microbial contamination in the air and archival entities in the warehouse
- Dermatophagoid mite
- Archival pests (such as tobacco armour, cockroaches, ants), rats, etc
- Outdoor air pollution
- Labor injury in the process of work
- Others

1. Do you know the health effects of occupational hazards in archival work?

- Yes
- No

1. Do you know how to effectively protect against the occupational hazards at work?

- Yes
- No

1. Has your unit issued any relevant documents on the protection of archival occupational hazard factors?

- Yes
- No

1. Have you done the protection work according to the occupational protection documents?

- Yes
- No

1. Is your unit equipped with the equipment and materials required for archival occupational protection?

- Yes
- No

1. Has your unit carried out archival occupational protection training?

- Yes
- No

ⅡIn the past 12 months, whether the following situation occurs, please fill in (questions 29 to 37).

1. Is there a mold smell in the workplace (except in the warehouse)?

- Yes, Every time you go in
- Yes, According to Season, mainly in summer and rainy season
- Never

1. Is there a moldy smell in the archives warehouse?

- Yes, Every time you go in
- Yes, According to Season, mainly in summer and rainy season
- Never

1. Is there a pungent chemical smell in the workplace?

- Yes, Every time you go in
- Yes, According to Season, mainly in summer and rainy season
- Never

1. Are there cockroaches, ants, tobacco beetles, bark beetles and other pests in the workplace?

- Yes, Every time you go in
- Yes, According to Season, mainly in summer and rainy season
- Never

1. Are there any signs of moisture in the workplace (multiple choice)?

- Yes, Every time you go in
- Yes, According to Season, mainly in summer and rainy season
- Never

1. Working hours in front of the computer

- ≥ 4 hours per day
- 1-3 hours per day
- 1 hour per day
- Never

1. The average number of times using printer and copiers

- ≥ 7 times per day
- 4-6 times per day
- 1-3 times per day
- Never

1. Have you ever had more frequent asthma or asthma attacks at work (especially when exposed to archival entities)?

- Appearance every time
- Often
- Once in a while
- Never

1. Does your family have respiratory genetic history (multiple choice)?

- Yes
- No
